# Association Between Serum Caffeine Concentrations, Intermittent Hypoxia and Apnea in Preterm Infants: A Prospective Observational Study

**DOI:** 10.3390/children13010085

**Published:** 2026-01-06

**Authors:** Gonca Vardar, Demet Oguz, Ilker Uslu, Sinem Gülcan Kersin, Merih Cetinkaya, Eren Ozek

**Affiliations:** 1 Department of Pediatrics, Division of Neonatology, School of Medicine, Marmara University, 34899 Istanbul, Türkiye; sinem.kersin@marmara.edu.tr (S.G.K.);; 2 Department of Pediatrics, Division of Neonatology, University of Health Sciences, Basaksehir Cam and Sakura City Hospital, 34480 Istanbul, Türkiye; demet.oguz@sbu.edu.tr (D.O.); sait.uslu@acibadem.com (I.U.);

**Keywords:** caffeine concentration, preterm infant, intermittent hypoxia, apnea

## Abstract

**Highlights:**

**What are the main findings?**

- Serum caffeine concentrations did not differ significantly across gestational age groups (23–27 vs. 28–30 weeks) during the postnatal period.
- Lower serum caffeine levels were associated with an increased frequency of apnea of prematurity and intermittent hypoxia episodes, particularly after the second week of life.

**What are the implications of the main finding?**

- Routine caffeine dose adjustment or serum monitoring based solely on gestational age may not be necessary in preterm infants ≤ 30 weeks’ gestation.
- Ensuring adequate serum caffeine concentrations may be important for reducing apnea and intermittent hypoxia, supporting individualized clinical assessment in symptomatic infants.

**Abstract:**

Background/Objectives: Caffeine citrate represents the standard pharmacological intervention for apnea of prematurity (AOP) and episodes of intermittent hypoxia (IH). Despite its widespread use, consensus regarding the necessity of routine serum monitoring, optimal dosing protocols, and precise clinical indications remains elusive. The primary objective of this investigation was to evaluate the longitudinal trajectory of serum caffeine concentrations in preterm infants and to analyze their correlation with the incidence of AOP and IH episodes. Furthermore, we sought to determine whether blood caffeine concentrations varied significantly across gestational ages throughout the postnatal period. Methods: This multicenter, prospective observational study enrolled preterm infants with a gestational age of ≤30 weeks. Participants were administered a standard loading dose of caffeine citrate within the first 24 h of life, followed by a standardized maintenance regimen. Serum caffeine levels were quantified on a weekly basis. The cohort was stratified into two distinct groups based on gestational age: Group 1 (23–27 weeks) and Group 2 (28–30 weeks). Results: The study yielded 588 serum caffeine measurements from a cohort of 104 preterm infants, characterized by a median gestational age of 28 weeks (range: 23–30 weeks) and a mean birth weight of 1034 ± 296 g. Statistical analysis revealed no significant disparities in serum caffeine concentrations across gestational age groups (*p* > 0.05). Notably, during the third week of life, infants with apneic episodes demonstrated significantly lower caffeine levels than those without apnea (*p* = 0.016). Furthermore, a significant negative correlation was identified between serum caffeine concentrations and the frequency of IH episodes during the third, fourth, and fifth weeks of life across multiple oxygen saturation thresholds. Conclusions: While serum caffeine concentrations in preterm infants did not vary significantly with gestational age, lower levels were associated with a higher incidence of AOP and IH episodes. These results suggest that while routine monitoring or dose adjustment based solely on gestational age may not be warranted, maintaining adequate serum levels is critical for symptom management. Future research should prioritize randomized controlled trials with expanded sample sizes, extended follow-up periods, and a rigorous analysis of adverse effects.

## 1. Introduction

Caffeine citrate functions as a robust respiratory stimulant, effectively diminishing the frequency of apneic events by alleviating or resolving the clinical manifestations of apnea of prematurity (AOP) [1]. Respiratory instability in this population frequently precipitates episodes of intermittent hypoxia (IH) and apnea, characterized by significant fluctuations in oxygenation [2]. AOP occurs in almost all infants below 28 weeks of gestational age, closely related to the degree of immaturity at birth. In addition, these infants have an unstable upper airway, which can lead to obstruction, and a soft chest wall, causing ventilatory instability [3]. Since IH episodes may contribute to the risk of neurodevelopmental impairment, severe retinopathy of prematurity (ROP), and bronchopulmonary dysplasia (BPD), this study focuses on the caffeine levels in the prevention of AOP and IH episodes [2]. Consequently, caffeine has been extensively adopted in neonatal intensive care units for the management of IH episodes and AOP, owing to its capacity to centrally stimulate respiratory drive via non-specific inhibition of adenosine receptors [4].

The landmark Caffeine for Apnea of Prematurity (CAP) trial utilized a standardized protocol comprising a 20 mg/kg loading dose of caffeine citrate followed by a 5–10 mg/kg maintenance dose. This trial established the long-term efficacy of caffeine, highlighting improved survival rates without neurodevelopmental disability at 18–21 months, as well as reductions in (BPD) and severe ROP. However, the original CAP trial did not specifically address the optimization of caffeine dosing or the relevance of serum concentrations [5].

The pharmacokinetics of caffeine in preterm infants are complex, with a half-life ranging from 72 to 96 h up to 40–230 h. The precise therapeutic threshold remains a subject of debate, complicated by factors such as hepatic enzyme maturation, inter-individual metabolic variability, and adenosine gene polymorphisms [6,7,8]. While therapeutic drug monitoring is generally deemed unnecessary, there are documented instances of toxic serum caffeine concentrations in infants receiving standard dosing regimens (10 mg/kg) [9].

Historically, serum caffeine concentrations in preterm infants have been maintained between 5 and 20 mg/L [10]. However, the literature suggests that dosing adjustments based on postnatal and gestational age may be beneficial. Over the past four decades, the recommended therapeutic range has shifted upward from 5 to 15 mg/L to 8–20 mg/L [11]. Furthermore, minimal serum concentrations required for effective AOP treatment have been suggested to lie within the 15–20 mg/L range [12].

Given these uncertainties, this study aimed to evaluate the longitudinal profile of serum caffeine levels in preterm infants and to assess their association with apnea and IH episodes. Additionally, we examined whether blood caffeine concentrations varied significantly across different gestational ages during the postnatal weeks and evaluated their relationship with other short-term clinical outcomes.

## 2. Materials and Methods

### 2.1. Study Design and Patient Population

This multicenter, prospective observational cohort study was conducted between September 2021 and September 2023 across the neonatal intensive care units of two tertiary centers in Istanbul, Türkiye: Marmara University Hospital and Basaksehir Cam and Sakura City Hospital. The research protocol received formal approval from the Marmara University Ethics Committee (3 September 2021; No: 09.2021.968).

The study population comprised preterm neonates born at a gestational age (GA) of ≤30 weeks who were indicated for early caffeine therapy. Participants were stratified into two cohorts based on gestational maturity: Group 1, consisting of extremely preterm infants (<28 weeks GA); and Group 2, comprising early preterm infants (≥28 weeks GA).

Exclusion criteria were defined as follows: (1) transfer to an external facility prior to the 30 weeks postmenstrual age; (2) presence of major congenital or chromosomal anomalies; (3) diagnosis of central nervous system disorders or congenital myopathies; (4) complex congenital heart defects; (5) thoracic abnormalities, such as chest wall deformities or diaphragmatic hernia; (6) hydrops fetalis; and (7) incomplete data acquisition during the study interval.

### 2.2. Study Protocol

All eligible neonates received an intravenous loading dose of caffeine citrate (20 mg/kg) administered via the umbilical vein within the first 24 h of life. Maintenance therapy commenced with a dosage of 5 mg/kg, which was titrated up to 10 mg/kg contingent upon the frequency of apnea and IH episodes. Dosing was adjusted weekly to account for weight gain once the infant surpassed their birth weight.

Serum caffeine concentrations were quantified using an enzyme-linked immunosorbent assay (ELISA) kit (My Bio Source, Inc., San Diego, CA, USA), requiring 0.1–0.5 mL of plasma. Blood specimens were drawn as trough levels, collected one hour prior to the administration of the maintenance dose. The initial sample was obtained at least 72 h after the loading dose, with subsequent sampling performed at weekly intervals. Caffeine pharmacotherapy was weaned and discontinued once infants demonstrated clinical stability, defined as the absence of positive pressure respiratory support for five consecutive days without apneic events.

Comprehensive demographic, perinatal, and neonatal data were recorded for analysis. Participants were monitored for potential adverse effects associated with caffeine, specifically tachycardia and hypertension. Hepatic and renal dysfunction were clinically defined based on biochemical markers: hepatic dysfunction as aspartate aminotransferase (AST) and/or alanine aminotransferase (ALT) levels > 70 U/L, or direct bilirubin > 1.0 mg/dL; and renal dysfunction as serum creatinine > 1.2 mg/dL or urine output < 1 mL/kg/h recorded on the day preceding serum caffeine analysis [13].

Following enrollment, arterial oxygen saturation (SpO2) was continuously monitored using bedside monitors (IntelliVue Mx550, Philips Healthcare, Böblingen, Germany) with pulse oximetry sensors (Nellcor Neonatal-Adult SpO2 Sensor, Covidien, Tullamore, Ireland). IH episodes were quantified via histograms representing percentages of each 24 h period, with the mean daily rate calculated weekly.

An IH episode was defined as a transient reduction in SpO2 below 90% lasting ≥5 s. These events were stratified by severity into three desaturation intervals: 85–90%, 80–85%, and <80%. Severe IH episodes were categorized as desaturations below 80% persisting for ≥5 s [2]. Apnea was clinically defined as a cessation of breathing exceeding 20 s, or shorter pauses accompanied by bradycardia (<100 bpm) or cyanosis (oxygen desaturation < 90%) [6].

### 2.3. Outcomes

The primary outcome was the correlation between serum caffeine concentrations and the frequency of apnea and IH episodes in infants ≤ 30 weeks GA. Secondary outcomes included longitudinal variation in serum caffeine levels relative to gestational age and the incidence of neonatal morbidities, specifically BPD, patent ductus arteriosus (PDA), intraventricular hemorrhage (IVH), necrotizing enterocolitis (NEC), and ROP. Diagnostic criteria utilized included the National Institute of Child Health and Human Development definitions for BPD [14], Volpe’s classification for IVH [15], and standardized international and Bell’s criteria for ROP and NEC, respectively [16,17].

### 2.4. Sample Size

Sample size estimation was conducted using G*Power software 3.1.9.6 to determine the effects of caffeine concentrations on AOP and IH episodes in preterm infants. A power analysis determined that a total sample size of 88 subjects was required to detect an effect size of 0.3 with 90% power at a 5% significance level. Ethical constraints precluded the inclusion of a placebo control group.

### 2.5. Statistical Analysis

Data analysis was executed using PASW Statistics 18.0 for Windows. Categorical variables were presented as frequencies and percentages [n (%)], while continuous variables were expressed as mean ± standard deviation (SD) or median with range (25–75% quartiles), depending on distribution. Normality was assessed via the Kolmogorov–Smirnov test. Group comparisons for continuous data were performed using the Mann–Whitney U test. Spearman’s rank correlation coefficient was employed to evaluate associations between serum caffeine concentrations and clinical variables. The Friedman test was utilized to analyze longitudinal paired data regarding weekly caffeine levels. Statistical significance was defined as a *p*-value < 0.05.

## 3. Results

### 3.1. Study Population and Demographics

**Figure 1:**
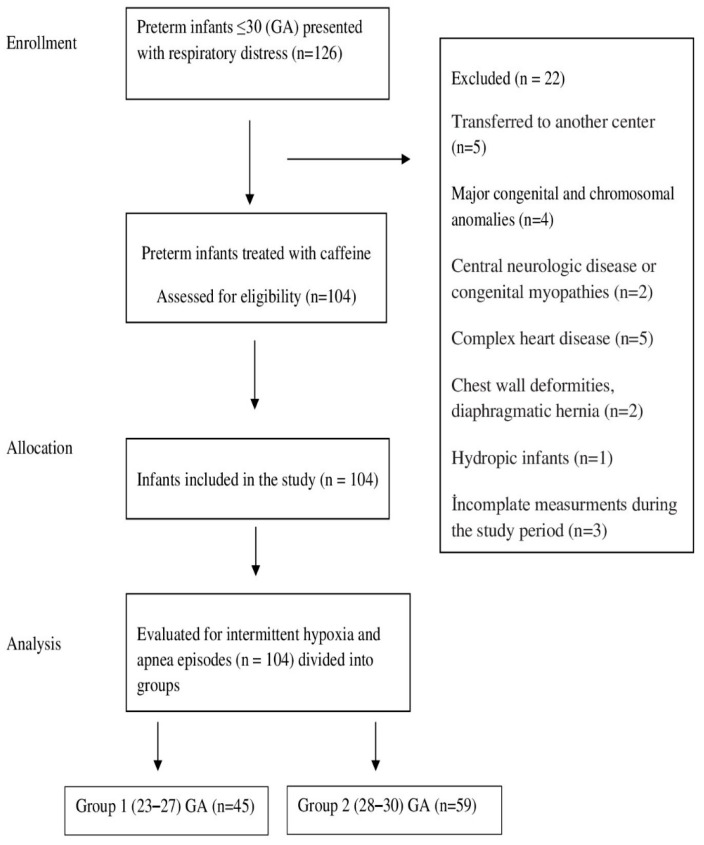
Flow chart for selection of eligible infants in the study GA; Gestational age.

The study cohort consisted of 104 preterm infants, characterized by a median gestational age of 28 weeks (range: 23–30 weeks) and a mean birth weight of 1034 ± 296 g. The population was stratified into two gestational age categories: Group 1 < 28 weeks, comprising 45 infants (43%), and Group 2 ≥ 28 weeks, comprising 59 infants (57%). Detailed demographic and clinical profiles are presented in Table 1. Over the course of the study, a total of 588 serum samples were analyzed, distributed as 335 samples from Group 1 and 253 from Group 2 (Figure 1).

### 3.2. Clinical Characteristics and Confounding Variables

Significant clinical disparities were observed between the two groups. Group 1 exhibited significantly lower Apgar scores at 1st and 5th minutes (*p* < 0.001) and a higher prevalence of maternal chorioamnionitis (*p* < 0.001). Furthermore, neonates in Group 1 had increased requirements for delivery room resuscitation (*p* < 0.001) and higher rates of respiratory distress syndrome (RDS) (*p* < 0.001), ventilator-associated pneumonia (*p* = 0.001), hemodynamically significant patent PDA (*p* < 0.001), and BPD (*p* = 0.001). Consequently, postnatal steroid administration (*p* < 0.001) and tachycardia (*p* = 0.003) were more frequent in this group, alongside a longer duration to discharge (*p* < 0.001).

Despite these differences, univariate analysis within Group 1 revealed no significant association between weekly serum caffeine levels and chorioamnionitis, ventilator-associated pneumonia, or postnatal steroid administration (*p* > 0.05). Multivariate logistic regression analysis, adjusting for confounding variables including chorioamnionitis, RDS, ventilator-associated pneumonia, hemodynamically significant PDA, and postnatal steroid usage, confirmed that these factors did not significantly correlate with weekly caffeine concentrations.

**Figure 2:**
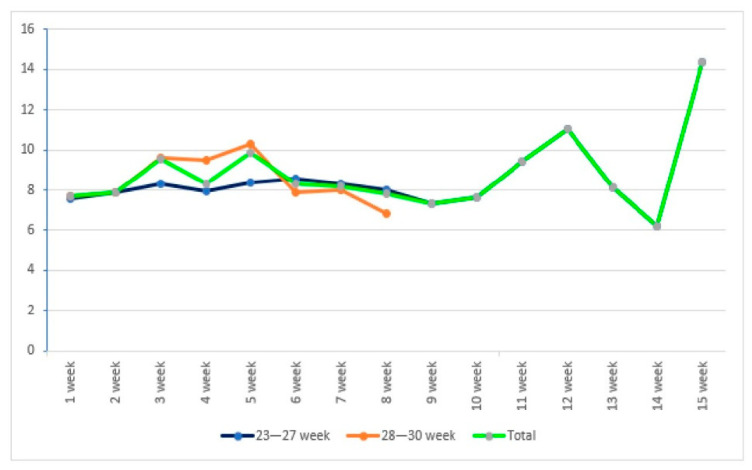
Serum caffeine concentrations according to gestational age of infants (mg/L).

Serum caffeine profiles, resulting from the standardized regimen of a 20 mg/kg loading dose followed by 5–10 mg/kg maintenance, were analyzed across gestational age groups (Figure 2, Table 2) and weight categories (<1000 g vs. >1000 g) (Table 3). Statistical analysis indicated no significant differences in serum caffeine concentrations across gestational age or body weight. Furthermore, longitudinal analysis demonstrated that serum caffeine levels did not fluctuate significantly across the postnatal weeks as gestational age increased (*p* > 0.05).

When comparing infants with and without AOP, a notable divergence in serum caffeine levels was identified. During the third week of life, infants experiencing apnea exhibited significantly lower serum caffeine concentrations compared to those who remained apnea-free (*p* = 0.016) (Table 4). However, no significant difference in caffeine levels was detected between AOP and non-AOP patients when aggregated across both groups generally (*p* > 0.05). Additionally, a subset analysis of neonates receiving non-invasive ventilation at the time of sampling revealed no significant difference in caffeine concentrations between those with and without apneic events (Table 5).

A consistent negative correlation was observed between serum caffeine levels and the frequency of IH episodes. During the 3rd, 4th, and 5th weeks of life, higher serum caffeine concentrations were significantly associated with a reduction in IH episodes across various saturation thresholds: SpO2 < 80% (r = 0.249, *p* = 0.018; r = 0.451, *p* < 0.001; r = 0.269, *p* = 0.041 for respective weeks), SpO2 80–85% (r = 0.345, *p* = 0.001; r = 0.328, *p* = 0.004; r = 0.265, *p* = 0.044) and SpO2 85–90%: (r = 0.226, *p* = 0.032; r = 0.306, *p* = 0.007; r = 0.289, *p* = 0.028) (Table 6)

Subgroup analyses further clarified these relationships (Table 7). In Group 1, IH episodes at the 4th week were negatively correlated with serum levels (SpO2 < 80%, r = 0.420, *p* = 0.009; SpO2 80–85%, r = 0.367, *p* = 0.023). In Group 2, negative correlations were evident at the 3rd week (SpO2 80–85%, r = 0.394, *p* = 0.005), 4th week (SpO2 < 80%, r = 0.434, *p* = 0.006), and 5th week (SpO2 < 80%, r = 0.445, *p* = 0.029; SpO2 80–85%, r = 0.488, *p* = 0.016; SpO2 85–90%, r = 0.474, *p* = 0.019). The median postmenstrual age for caffeine withdrawal was 35 weeks (range: 25–40 weeks).

Regarding adverse effects, infants exhibiting tachycardia (n = 12) presented with significantly higher serum caffeine concentrations at the 8th week compared to those without tachycardia (n = 92) [14.85 (11.05–17.65) vs. 7.37 (6.7–13.6) mg/L, *p* = 0.045], although these levels remained within the therapeutic range. The majority of tachycardic infants (83.3%) were in Group 1. No significant associations were found regarding feeding intolerance or hypertension. Additionally, no statistically significant differences in serum caffeine levels were observed between infants with and without hepatic or renal dysfunction, defined by AST/ALT > 70 U/L, direct bilirubin > 1.0 mg/dL, or creatinine > 1.2 mg/dL.

Analysis of neonatal morbidities indicated no significant difference in serum caffeine levels for infants with BPD, NEC, PDA, or ROP across both groups (*p* > 0.05). However, in the first week of life, infants in Group 1 diagnosed with IVH (n = 14) had significantly higher caffeine concentrations compared to those without IVH [7.85 (6.2–23.6) vs. 7.4 (6–15.4) mg/L, *p* = 0.04]. A positive correlation was identified between serum caffeine levels and the presence of IVH in Group 1 during this period (r = 0.243, *p* = 0.022). Within the IVH cohort, no significant difference in caffeine levels was found between lower grades (1–2, n = 6) and severe grades (3–4, n = 8) [7.7 (6–16.4) vs. 7.7 (6.2–23.6) mg/L, *p* = 0.642].

## 4. Discussion

Whether serum caffeine levels vary according to gestational age and their clinical significance in reducing AOP and IH episodes are questions that need to be answered. Caffeine citrate, a methylxanthine derivative, serves as the cornerstone of pharmacotherapy for AOP owing to its prolonged half-life, cost-effectiveness, and demonstrated neuroprotective benefits. While the precise mechanism of action remains partially elucidated, its respiratory efficacy is primarily attributed to the non-specific antagonism of adenosine receptors. This blockade stimulates the brainstem respiratory centers, resulting in enhanced minute ventilation, increased skeletal muscle tone, heightened sensitivity to carbon dioxide, and an elevated metabolic rate coupled with increased oxygen consumption [3].

Pharmacokinetically, caffeine metabolism exhibits distinct developmental variations. In adults, the drug is primarily metabolized and cleared via the hepatic CYP1A2 enzyme system. Conversely, preterm infants demonstrate immature CYP1A2 activity and diminished renal tubular reabsorption; consequently, caffeine is mostly excreted unchanged in the urine. Since the incidence of chorioamnionitis was significantly higher in Group 1, it can be inferred that inflammation affects caffeine metabolism by down-regulating hepatic and extrahepatic cytochrome enzymes. Although chorioamnionitis was not found to be a significant cofounding variable of caffeine levels in our study, changes in caffeine metabolism due to multiorgan involvement in infection may have an effect on caffeine metabolism [18]. Historically, this physiological immaturity led to the hypothesis that lower GA and reduced glomerular filtration rates would precipitate higher serum caffeine concentrations [9]. However, the data from this study contradict that assumption, revealing no statistically significant correlation between serum caffeine concentrations and postmenstrual age, body weight, or markers of renal and hepatic dysfunction. These findings suggest that caffeine pharmacokinetics in this population are influenced by a complex interplay of physiological factors beyond gestational maturity alone [8].

Despite widespread caffeine use, consensus on optimal dosing strategies remains elusive. The pivotal CAP trial established that early administration (within the first 10 days) significantly reduced the duration of mechanical ventilation and the incidence of BPD [19]. However, the CAP trial did not investigate optimal loading or maintenance doses, nor did it address target serum concentrations. Clinically, neonatologists typically titrate dosages based on therapeutic response—increasing the dose during apneic events and reducing it upon the emergence of toxicity [20]. Our study highlights a significant association: infants experiencing AOP during the third week of life had significantly lower serum caffeine levels than asymptomatic controls. Undoubtedly, this difference was not observed in infants receiving non-invasive ventilation, which has an apnea-reducing effect, suggesting that the relationship between serum levels and clinical outcomes is multifaceted and potentially modulated by genetic factors, such as adenosine and dopamine receptor polymorphisms [21,22].

IH, defined as cyclical desaturation (SpO2 < 90% for ≥5 s) followed by reoxygenation, represents a significant clinical challenge. Other notable definitions of IH episodes include a decline in SaO2 by ≥10% from baseline, lasting for ≥5 s, terminating with saturation increases above the event threshold [1,2,23]. These transient hypoxic events, even when not culminating in frank apnea, may contribute to cumulative organ hypoxia. Evidence from the Latte Dosage Trial indicates that caffeine prophylaxis in late preterm infants may mitigate IH episodes, thereby improving long-term prognosis [24]. Similarly, Rhein et al. reported that a 6 mg/kg maintenance dose reduced IH events at 35–36 weeks postmenstrual age [1]. Our findings corroborate these observations, demonstrating a negative correlation between serum caffeine levels and the frequency of IH episodes. Specifically, higher caffeine concentrations—even within the standard therapeutic range—were associated with reduced desaturation severity.

The optimal timing for caffeine discontinuation remains another area of debate. The American Academy of Pediatrics recommends cessation after a 5–7-day period free of cardiorespiratory events, typically around 34–35 weeks postmenstrual age [6]. In our cohort, the median age of withdrawal was 35 weeks. Whether extending therapy exerts a prophylactic effect against residual IH episodes requires further investigation.

Safety remains a paramount concern. Common adverse effects of caffeine therapy include tachycardia, feeding intolerance, and hypertension [25]. While some studies describe tachycardia as a mild, transient side effect often leading to unnecessary therapy cessation [26], our data revealed a specific association. At the eighth week, infants with tachycardia exhibited significantly higher serum caffeine concentrations—specifically in lower GA infants—although these levels generally remained within the accepted therapeutic window of 8–20 mg/L [9,13].

Regarding major morbidities, the CAP trial reported protective effects of caffeine against severe ROP and reduced requirements for PDA closure [5,25]. Conversely, other investigations have suggested potential risks, such as pulmonary over-circulation or aggressive posterior ROP associated with prolonged therapy [27,28]. These conflicting results may be due to differences in caffeine dose or patient characteristics [8]. Our study found no correlation between serum levels and the incidence of PDA, NEC, or ROP. However, we observed a statistically significant association between higher caffeine concentrations and the incidence of IVH in Group 1 during the first week of life. While the grade of IVH did not correlate with drug levels, this finding warrants caution. Preclinical models suggest that high caffeine exposure may impair myelination and glial cell proliferation [29,30], and a randomized trial of high-dose caffeine in preterm infants reported an increased risk of cerebellar hemorrhage [31]. Therefore, the link between early high serum levels and IVH observed here should be interpreted as an association requiring validation in larger cohorts.

The primary strength of this study lies in its prospective design, which involves weekly serum quantification in a vulnerable, extremely low birth weight population. However, limitations exist. Pulse oximetry cannot distinguish between central and obstructive etiologies for apnea and IH episodes, and the absence of esophageal pressure monitoring and respiratory inductive plethysmography precluded precise phenotypic characterization. Additionally, while the study was multicenter, variations in general neonatal care could act as confounders. Finally, the lack of long-term neurodevelopmental follow-up limits conclusions regarding the clinical impact of the observed biochemical associations.

## 5. Conclusions

This study demonstrates that serum caffeine concentrations in preterm infants do not vary significantly with gestational age alone. However, lower serum caffeine levels were significantly associated with an increased incidence of AOP and IH episodes. While these findings underscore the clinical relevance of maintaining adequate serum levels for symptom control, they do not currently support deviating from standard protocols toward routine therapeutic drug monitoring based solely on GA. Future randomized controlled trials with larger sample sizes are essential to define optimal therapeutic windows that balance symptom management with safety, particularly concerning neurological outcomes.

## Figures and Tables

**Table 1:** Demographic and Clinical Characteristics of the Study Cohort.

Characteristics	23–27 GA (n = 45)	28–30 GA (n = 59)	*p*
Birth weight, g	810 (700–1000)	1255 (972–1385)	**<0.001 ^a^**
Gender, male %	20 (44.4)	33 (55.9)	0.246 ^b^
Casarean delivery, %	33 (73.3)	49 (83.1)	0.229 ^b^
Apgar 1 min median (min–max)	4 (3–5)	5 (4–7)	**<0.001 ^a^**
Apgar 5 min median (min–max)	6 (5–7)	7 (6–8)	**<0.001 ^a^**
PPROM with chorioamnionitis, %	10 (22.2)	0	**<0.001 ^b^**
Prenatal 2 doses steroids, %	24 (53.3)	35 (60.3)	0.476 ^b^
GDM, %	2 (4.4)	4 (6.8)	0.613 ^b^
Resuscitation at delivery room, %	39 (86.7)	29 (49.2)	**<0.001 ^b^**
Preeclampsia, %	14 (68.9)	18 (69.5)	0.947 ^b^
RDS, %	37 (82.2)	28(47.5)	**<0.001 ^b^**
VAP,%	8 (17.8)	0	**0.001 ^b^**
Postnatal steroid %	36 (80)	15 (25.4)	**<0.001 ^b^**
Tachycardia, %	10 (22.2)	2 (3.4)	**0.003 ^b^**
Hypertension %	2 (1.9)	1(1)	0.407
Feeding intolerance, %	18 (40)	13 (22)	0.05 ^b^
hsPDA, %	25 (55.6)	10(16.9)	**<0.001 ^b^**
Surgical ligation	3	0	*
BPD, %	37 (86)	23 (39)	**0.001 ^b^**
Discharge day, median	106 (70–132)	44 (35–59)	**<0.001 ^a^**
Mortality, %	2 (4.4)	1 (1.7)	0.407 ^b^

^a^ Mann–Whitney U test, ^b^ chi-Square test. Statistically significant values are shown in bold in the table (*p* < 0.05). GA, gestational age; PPROM, preterm premature rupture of membranes; GDM, gestational diabetes mellitus; RDS, respiratory distress syndrome; VAP, ventilator-associated pneumonia; hsPDA, hemodynamically significant patent ductus arteriosus; BPD, bronchopulmonary dysplasia. * Statistical comparison was not performed due to insufficient sample size.

**Table 2:** Serum caffeine concentrations (mg/L) according to gestational age of infants.

Weeks	N	23–27 GA (n = 45)	N	28–30 GA (n = 59)	N	Total	*p*
1	45	7.6 (6.7–9.6)	59	7.7 (6.5–10.7)	104	7.7 (6.6–10.35)	0.713
2	45	7.9 (6.5–10.6)	59	7.9 (7–11.5)	104	7.9 (6.53–11.05)	0.443
3	41	8.3 (6.5–13)	49	9.6 (7.2–13.7)	90	9.52 (6.8–13.5)	0.248
4	38	7.95 (6.3–12.9)	39	9.5 (7.56–12.6)	77	8.3 (6.92–12.7)	0.183
5	34	8.4 (6.5–12.8)	26	10.25 (7.1–12.2)	60	9.88 (6.6–12.4)	0.370
6	32	8.55 (6.7–12.5)	16	7.88 (6.25–12.7)	48	8.3 (6.5–12.65)	0.801
7	26	8.33 (6.7–14.7)	9	8 (6.78–10.4)	35	8.17 (6.7–13.45)	0.487
8	22	7.99 (6.79–14.1)	3	6.8 (6.7–7.5)	25	7.8 (6.79–13.9)	0.259
9	15	7.3 (6.52–11.5)	0		15	7.3 (6.52–11.5)	***
10	11	7.65 (6.77–14.9)	0		11	7.65 (6.77–14.9)	***
11	8	9.42 (6.28–17.3)	0		8	9.42 (6.28–17.3)	***
12	8	11 (7.06–17.75)	0		8	11 (7.06–17.75)	***
13	4	8.11 (7.47–11.59)	0		4	8.11 (7.47–11.59)	***
14	2	6.23 (6.13–6.33)	0		2	6.23 (6.13–6.33)	***
15	2	14.35 (6.07–22.62)	0		2	14.35 (6.07–22.62)	***

GA, gestational age. *** Statistical comparison was not performed due to insufficient sample size.

**Table 3:** Serum caffeine concentrations (mg/L) according to weight.

Weeks	N	<23–1000g (n = 55)	N	>1000g (n = 49)	N	Total	*p*
1	55	7.6 (6.6–10.2)	49	7.8 (6.8–11.3)	104	7.7 (6.6–10.35)	0.454
2	47	8 (6.4–11.1)	57	7.8 (6.8–10.8)	104	7.9 (6.53–11.05)	0.822
3	30	8.95 (6.7–13.5)	60	9.52 (6.8–13.5)	90	9.52 (6.8–13.5)	0.540
4	19	7.75 (6.3–13.4)	57	9 (7.4–12.7)	76	8.3 (6.92–12.7)	0.153
5	9	8.8 (7–10)	49	9.95 (6.6–12.3)	58	9.88 (6.6–12.4)	0.621
6	2	12.25 (8–16.5)	45	8.1 (6.4–12.4)	47	8.3 (6.5–12.65)	0.292
7	1	14.8 (14.8–14.8)	34	8 (6.7–13.2)	35	8.17 (6.7–13.45)	0.229

**Table 4:** Serum caffeine levels (mg/L) of infants with and without apnea.

Weeks	N	Non-Apnea	N	Apnea	N	Total	*p*
1	58	7.4 (6.5–9.7)	46	7.8 (6.94–10.5)	104	7.7 (6.6–10.35)	0.212
2	58	7.8 (6.7–10.8)	46	7.95 (6.5–11.1)	104	7.9 (6.53–11.05)	0.948
3	46	10.2 (7.3–14.1)	44	8 (6.25–11.9)	90	9.5 (6.8–13.5)	**0.016**
4	35	9.5 (6.92–15.2)	42	8.02 (6.7–12.1)	77	8.3 (6.92–12.7)	0.223
5	27	10 (6.5–13.1)	33	9.8 (6.9–12.2)	60	9.88 (6.6–12.4)	0.841
6	19	8.5 (6.6–13.4)	29	8.1 (6.3–12.1)	48	8.3 (6.5–12.65)	0.555
7	12	9.3 (6.4–13.45)	23	8 (6.75–13.15)	35	8.17 (6.7–13.45)	0.801
8	6	10.4 (6.7–14.1)	19	7.8 (6.79–13.7)	25	7.8 (6.79–13.9)	0.874
9	4	8.05 (6.5–9.95)	11	7.3 (6.52–12.5)	15	7.3 (6.52–11.5)	0.514
10	2	9.05 (6.2–11.9)	9	7.65 (6.8–14.9)	11	7.6 (6.77–14.9)	0.637
11	1	22.1 (22.1–22.1)	7	8.33 (6.26–14.9)	8	9.4 (6.28–17.3)	0.127
12	1	20.4 (20.4–20.4)	7	9.8 (6.98–15.1)	8	11 (7.06–17.75)	0.275
13	0		4	8.11 (7.47–11.59)	4	8.1 (7.47–11.59)	***
14	0		2	6.23 (6.13–6.33)	2	6.2 (6.13–6.33)	***
15	0		2	14.35 (6.07–22.62)	2	14.3 (6.07–22.62)	***

*** Statistical comparison was not performed due to insufficient sample size.

**Table 5:** Serum caffeine levels (mg/L) of infants with apnea on non-invasive ventilation.

Weeks	N	Non-Apneic	N	Apneic	N	Total	*p*
1	28	7.45 (6.41–11.1)	17	9 (7.5–10.5)	45	8 (6.9–10.7)	0.349
2	20	7.95 (7.25–11.5)	23	7.6 (6.5–9.7)	43	7.9 (7–10.3)	0.223
3	12	10.55 (7.7–14.05)	23	8 (6.48–11.6)	35	9.45 (6.8–12.6)	0.122
4	9	10 (7.3–12.9)	24	7.95 (7.22–12.4)	33	8.03 (7.3–12.6)	0.544
5	8	6.9 (6.1–8.75)	18	8.4 (6.5–11.1)	26	8 (6.5–11)	0.210
6	8	6.99 (6.5–7.9)	19	8.1 (6.24–11.6)	27	7.75 (6.4–10.3)	0.326
7	6	8.19 (6.7–11)	16	7.47 (6.62–13.15)	22	7.47 (6.7–12.6)	0.740
8	3	6.97 (6.7–22.1)	9	7.8 (7.2–8)	12	7.59 (6.88–10.85)	0.644
9	2	6.5 (6.1–6.9)	10	8.65 (6.85–12.5)	12	7.2 (6.69–12)	0.132
10	1	6.2 (6.2–6.2)	7	8.11(6.77–16.2)	8	7.51 (6.48–15.55)	0.275
11	1	22.1 (22.1–22.1)	5	10.5 (6.26–14.9)	6	12.7 (6.26–19.7)	0.143
12	1	20.4 (20.4–20.4)	5	12.2 (9.8–15.1)	6	13.65 (9.8–20.4)	0.380
13	0		2	11.59 (8.19–15)	2	11.59 (8.19–15)	***
14	0		1	6.13 (6.13–6.13)	1	6.13 (6.13–6.13)	***
15	0		2	14.35 (6.07–22.62)	2	14.35 (6.07–22.62)	***

*** Statistical comparison was not performed due to insufficient sample size.

**Table 6:** Correlation of intermittent hypoxia episodes with serum caffeine levels in both groups.

Weeks		SpO2 < 80%	SpO2 80–85%	SpO2 80–85%
1	r	0.001	−0.001	−0.140
	*p*	0.995	0.991	0.155
	N	104	104	104
2	r	−0.140	−0.118	−0.055
	*p*	0.158	0.232	0.581
	N	104	104	104
3	r	−0.249	−0.345	−0.226
	*p*	**0.018**	**0.001**	**0.032**
	N	90	90	90
4	r	−0.451	−0.328	−0.306
	*p*	**0.000**	**0.004**	**0.007**
	N	76	76	76
5	r	−0.269	−0.265	−0.289
	*p*	**0.041**	**0.044**	**0.028**
	N	58	58	58
6	r	0.029	0.243	−0.016
	*p*	0.848	0.099	0.916
	N	47	47	47
7	r	0.020	0.137	0.105
	*p*	0.907	0.426	0.544
	N	36	36	36
8	r	0.061	0.214	0.102
	*p*	0.771	0.304	0.627
	N	25	25	25
9	r	0.518	0.222	0.123
	*p*	0.051	0.426	0.663
	N	15	15	15
10	r	0.401	0.000	0.167
	*p*	0.221	1.000	0.623
	N	11	11	11
11	r	0.639	0.430	0.317
	*p*	0.088	0.288	0.444
	N	8	8	8
12	r	0.412	0.221	−0.049
	*p*	0.310	0.599	0.907
	N	8	8	8
13	r		−0.316	−0.738
	*p*		0.684	0.262
	N		4	4

**Table 7:** Correlation of intermittent hypoxia episodes with serum caffeine levels in group 1 and group 2.

		Group 1			Group 2		
Weeks		SpO2 < 80%	SpO2 80–85%	SpO2 80–85%	SpO2 < 80%	SpO2 80–85%	SpO2 80–85%
1	r	0.031	0.122	−0.195	−0.036	−0.010	−0.080
	*p*	0.841	0.425	0.200	0.784	0.940	0.545
	N	45	45	45	59	59	59
2	r	−0.154	−0.010	0.075	−0.095	−0.148	−0.091
	*p*	0.312	0.951	0.625	0.474	0.263	0.495
	N	45	45	45	59	59	59
3	r	−0.163	−0.278	−0.087	−0.257	−0.394	−0.264
	*p*	0.307	0.078	0.588	0.075	**0.005**	0.067
	N	41	41	41	49	49	49
4	r	−0.420	−0.367	−0.272	−0.434	−0.271	−0.370
	*p*	**0.009**	**0.023**	0.099	**0.006**	0.099	**0.022**
	N	38	38	38	38	38	38
5	r	−0.169	−0.156	−0.186	−0.445	−0.488	−0.474
	*p*	0.340	0.379	0.292	**0.029**	**0.016**	**0.019**
	N	34	34	34	24	24	24
6	r	−0.124	0.313	0.192	0.321	0.139	−0.288
	*p*	0.498	0.081	0.293	0.243	0.621	0.299
	N	32	32	32	15	15	15
7	r	0.069	0.324	0.232	−0.279	−0.562	−0.533
	*p*	0.731	0.099	0.245	0.467	0.115	0.139
	N	27	27	27	9	9	9
8	r	0.152	0.270	0.137	−0.500	−0.866	−0.500
	*p*	0.498	0.224	0.543	0.667	0.333	0.667
	N	22	22	22	3	3	3
9	r	0.518	0.222	0.123			
	*p*	0.051	0.426	0.663			
	N	15	15	15			
10	r	0.401	0.000	0.167			
	*p*	0.221	1.000	0.623			
	N	11	11	11			
11	r	0.639	0.430	0.317			
	*p*	0.088	0.288	0.444			
	N	8	8	8			
12	r	0.412	0.221	−0.049			
	*p*	0.310	0.599	0.907			
	N	8	8	8			
13	r		−0.316	−0.738			
	*p*		0.684	0.262			
	N		4	4			

## Data Availability

The data presented in this study are available on request from the corresponding author. The data are not publicly available due to privacy or ethical restrictions.
